# Diagnosis and Management of Papillon-Lefevre Syndrome: A Rare Case Report and a Brief Review of Literature

**DOI:** 10.7759/cureus.43335

**Published:** 2023-08-11

**Authors:** Tanvi Phull, Divya Jyoti, Ritu Malhotra, Shanteri Nayak, Himanshi Modi, Ishu Singla, Aishwarrya P

**Affiliations:** 1 Department of Oral and Maxillofacial Surgery, Gian Sagar Dental College, Rajpura, Patiala, IND; 2 Department of Oral Health Sciences, Post Graduate Institute of Medical Education and Research (PGIMER) Chandigarh, Chandigarh, IND; 3 Department of Prosthodontics, ITS Centre for Dental Studies and Research, Ghaziabad, IND; 4 Department of Periodontology, Punjab Government Dental College and Hospital, Amritsar, Amritsar, IND; 5 Department of Orthodontics and Dentofacial Orthopedics, Sri Ramakrishna Dental College and Hospital, Coimbatore, IND

**Keywords:** genetic, hyperkeratosis, cathepsin c, ctsc gene, papillon-lefevre syndrome

## Abstract

Papillon-Lefevre syndrome (PLS) manifests as an autosomal recessive disorder caused by a mutation in the cathepsin C (CTSC) gene. This genetic alteration results in palmoplantar hyperkeratosis, rapid onset of periodontitis, and premature shedding of both primary and permanent teeth. The major etiological factor responsible for the development of this disorder appears to be variations in the CTSC gene, which is responsible for the production of the cathepsin C enzyme in the body. The multifactorial aetiology of the syndrome is influenced by immunologic, genetic, or microbial factors. This case report presents a clinical picture of a 21-year-old Indian male patient with oligodontia and mobile teeth accompanied by palmoplantar keratosis and a history of recurrent infection. The detailed family history of the patient revealed genetic relevance with PLS. This article will discuss in detail the diagnosis, evaluation and treatment modalities involved in the management of the case.

## Introduction

Papillon and Lefèvre, two French physicians, first described Papillon-Lefevre syndrome (PLS) in 1924, followed by the addition of another symptom of dural calcification in 1964 by Gorlin et al. [1]. It manifests as an autosomal recessive disorder caused by a mutation in the cathepsin C (CTSC) gene. The primary characteristics of this syndrome include early loss of permanent teeth, destructive periodontitis, diffuse palmoplantar keratoderma (PPK), and frequent pyogenic infections. PLS in the common population has an extremely low prevalence rate between 1/25,000 and 1/1,000,000 [2].

The susceptibility of PLS patients to periodontal diseases is increased on account of systemic conditions, including the endocrine, immune, and connective tissue status, which could promote rapid and severe loss of attachment [1]. The etiology of PLS varies from case to case and includes familial causes, acquisition due to other reasons, or association with systemic conditions. However, in most instances, the underlying cause can always be traced back to mutations in the alleles of the CTSC gene located on chromosome 11q14.2. The majority of patients with PLS exhibit homozygous CTSC mutations. In this article, a well-documented case report of a 21-year-old male patient who presented with PLS is studied in detail. The paper delves into a comprehensive examination of the oral and cutaneous symptoms associated with the syndrome, as well as the current treatment approaches used to manage the condition.

## Case presentation

A 21-year-old Indian male patient reported to the Department of Periodontics at Punjab Government Dental College, Amritsar, with a chief complaint of mobile teeth and pain in the upper and lower teeth region for the past five years. On acquiring detailed dental history through the patient’s parents, it was revealed that his deciduous teeth had begun to shed early by his fourth year. By the age of 13, multiple permanent teeth of the patient were exfoliated due to mobility.

**Figure 1:**
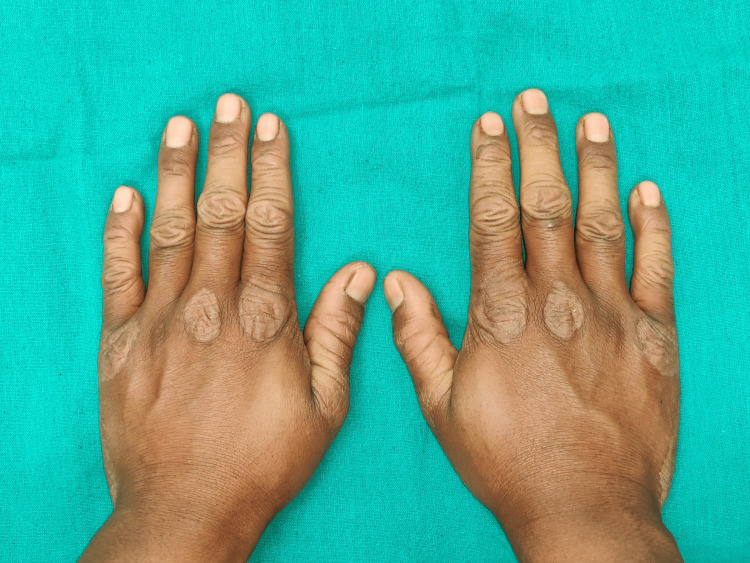
Hyperkeratosis on the dorsal surface of hand

**Figure 2:**
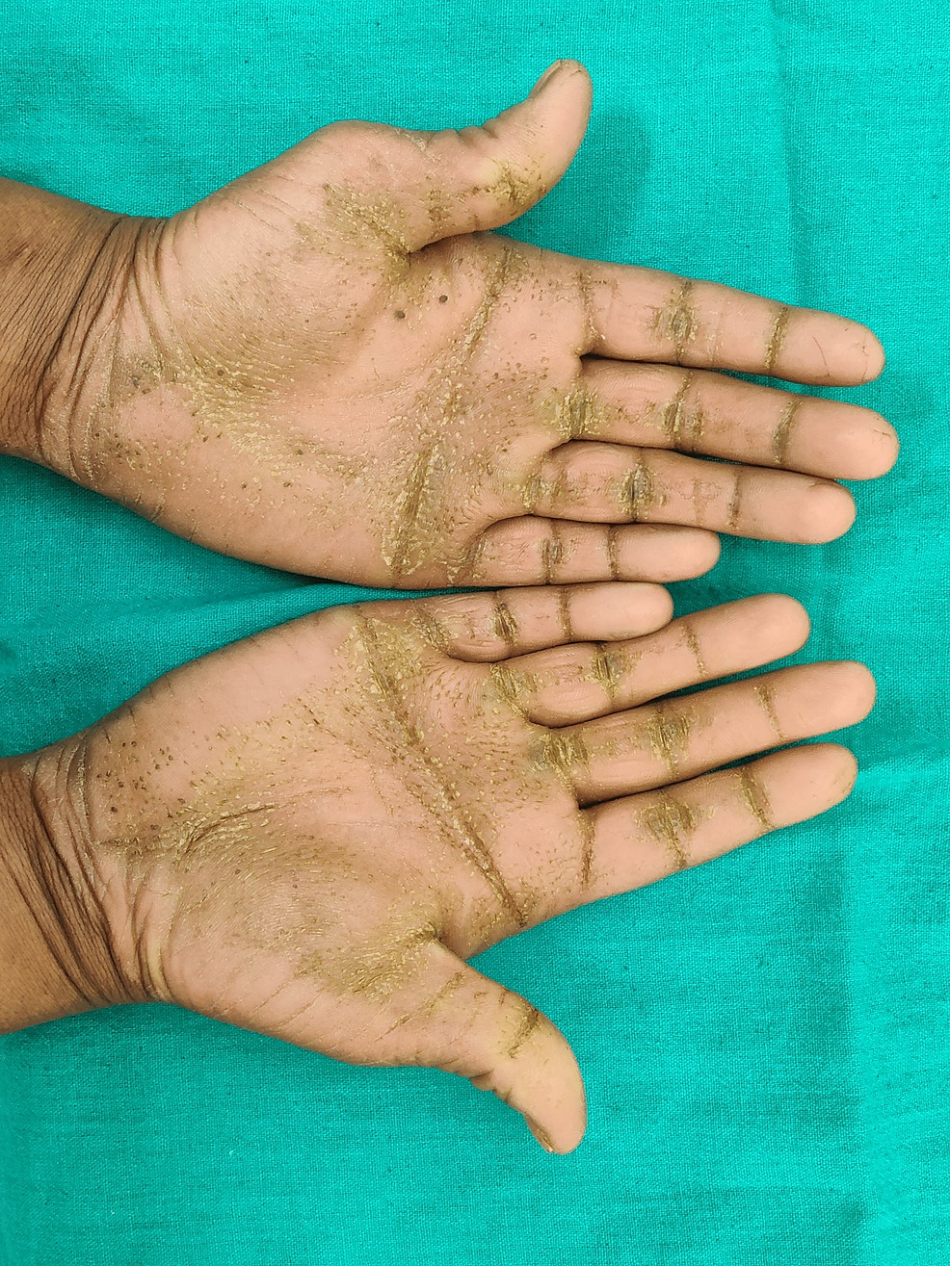
Hyperkeratosis on the ventral surface of hand

**Figure 3:**
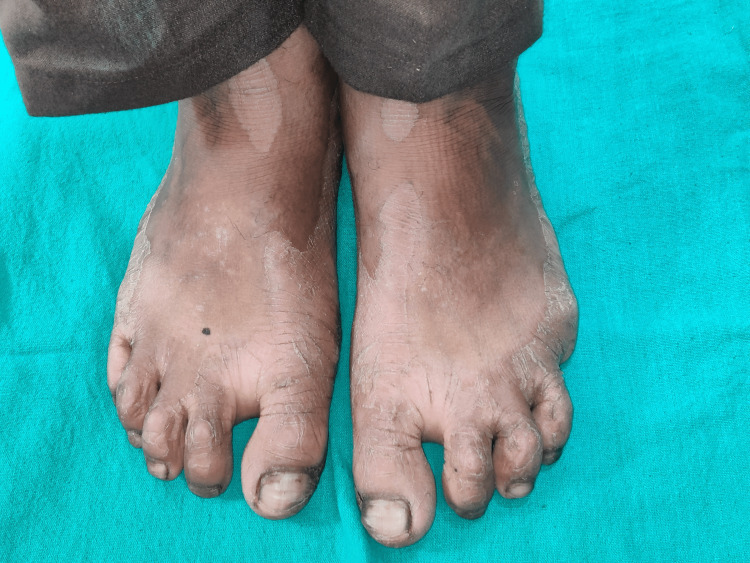
Hyperkeratosis on the dorsal surface of feet.

**Figure 4:**
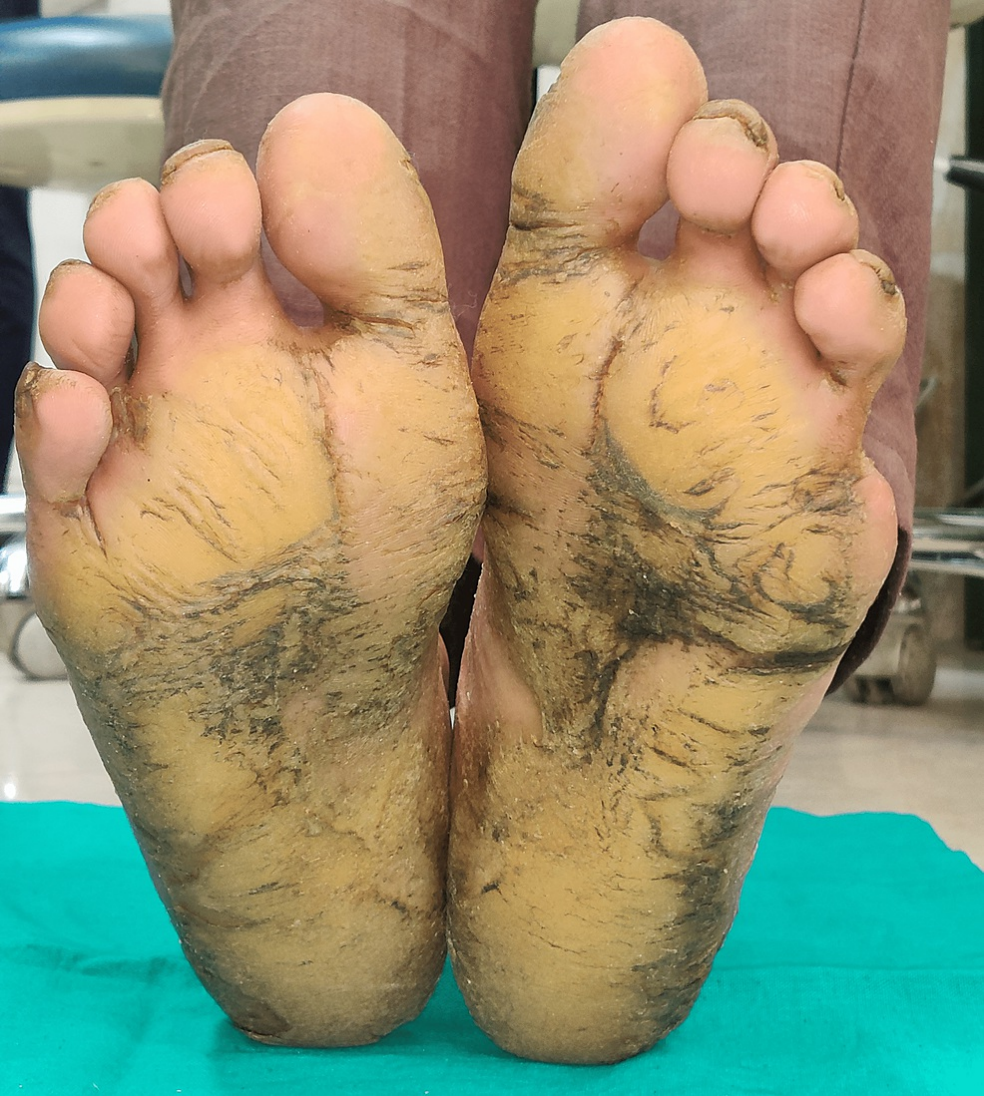
Hyperkeratosis on the ventral surface of feet

**Figure 5:**
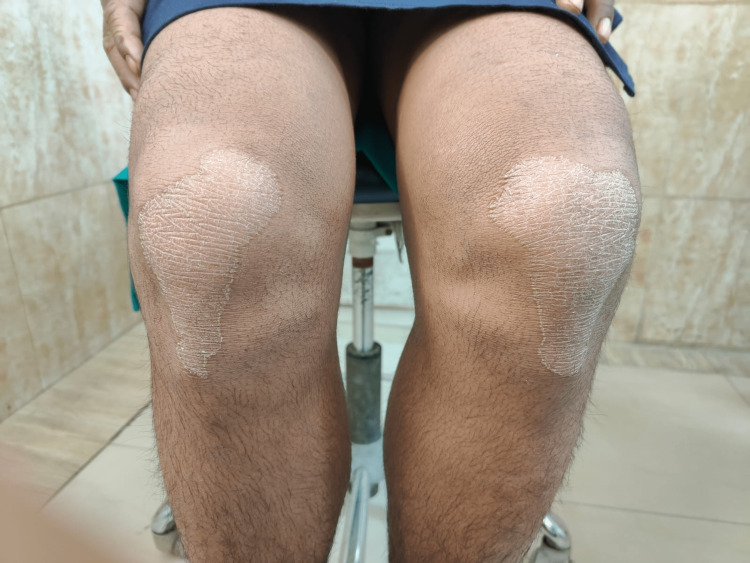
Hyperkeratosis on the dorsal surface of knee

The general medical history of the patient revealed a history of recurrent fever and infection, for which the patient was treated using intravenous (IV) fluids. The patient also had a history of recurrent dry scaling of the palm first observed at the age of seven months. On physical examination, bilateral palmar-plantar hyperkeratosis was observed with keratosis present on the dorsal and ventral surfaces of the hands (Figures 1, 2) and feet (Figures 3, 4) and the right and left knees (Figure 5). The patient has a missing toe in the left foot which has been reported lost due to an accident. Thickened index toenail was observed on both the left and right feet. A comprehensive family history revealed consanguineous marriages between the patient's parents and also other relatives in the family. The patient also reported of a cousin who had presented with similar clinical features and was rehabilitated for it.

**Figure 6:**
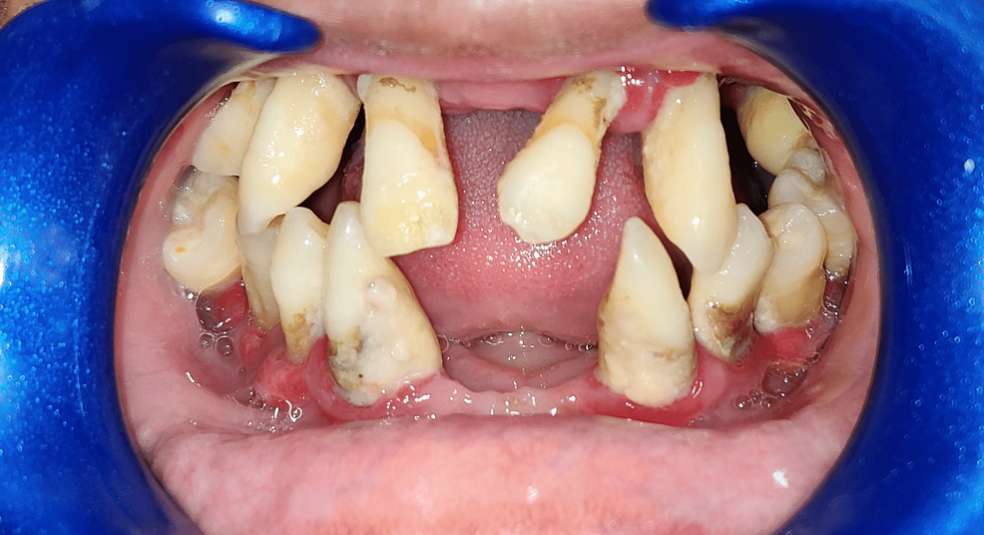
Intraoral photograph of missing permanent teeth in maxilla and mandible

During the intraoral examination, a total of 17 permanent teeth were observed including seven teeth in the maxillary arch and the remaining 10 in the mandibular arch (Figure 6). All teeth present displayed Miller's Grade III gingival recession, varying levels of flaring and Grade III mobility (as per Miller's Tooth Mobility Index 1950). Tissues of the gingiva were severely inflamed (Modified Gingival Index by Lobene, Weatherford, Ross. Lamm and Menaker 1986) and were tender on palpation. 

**Figure 7:**
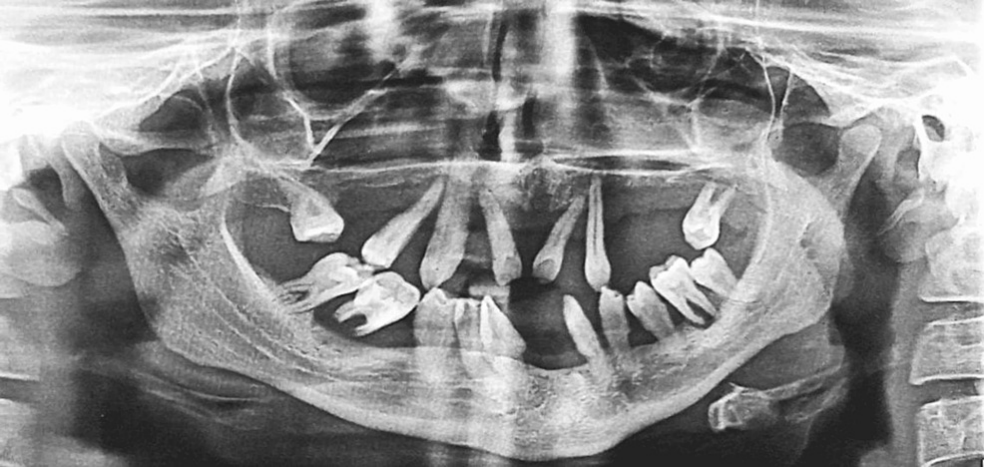
A panoramic radiograph of the oral cavity showing a floating-in-air appearance of teeth

A panoramic radiograph of the oral cavity displayed generalized horizontal alveolar bone loss and floating teeth with respect to the mandibular right and left first molars (Figure 7). The radiograph also revealed the absence of third molars along with multiple other permanent teeth. Based on the clinical features and evidence in the radiograph reports, two close differential diagnoses, Papillon- Lefevre Syndrome and Haim-Munk syndrome, were put together. Haim-Munk syndrome is an allelic variant of PLS and its clinical features, in addition to palmoplanter keratoderma and loss of dentition, include arachnodactyly (claw-like phalanges with convex nails) and acroosteolysis.

The patient’s routine haematological examination (complete blood count and viral markers) was found to be normal. The patient also underwent genetic testing at MedGenome Labs Ltd. (Bangalore, India), and the clinical exome analysis report showed a homozygous 5' splice site variation within intron 2 of the CTSC gene (chr11:g.88334936C>T; Depth:122x). This variation impacts the invariant GT donor splice site of exon 2 (c.318+1G>A; ENST00000227266.10). American College of Medical Genetics classifies this CTSC variation as pathogenic [3]. The genetic test reports led to confirming the provisional diagnosis.

**Figure 8:**
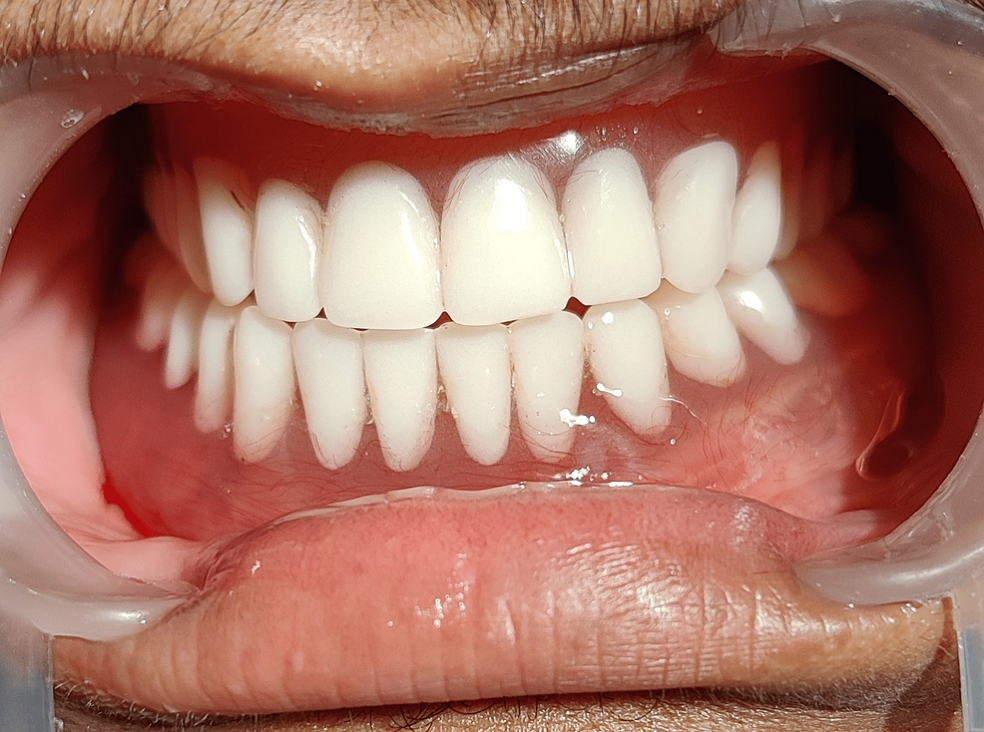
Prosthetic rehabilitation with an immediate complete denture

Treatment plans including emergency full mouth extraction, diet control, patient education and prosthetic rehabilitation using complete dentures or implant-supported overdentures were suggested. Written consents were obtained from both patient and his parents on making informed decisions for further treatments. The patient underwent phase II periodontal therapy or the non-surgical periodontal therapy (diet control and patient education in this case), following which all teeth with poor prognosis were extracted. Subsequently, the patient was presented with various treatment options to address his edentulous state. The patient favoured an immediate replacement in the form of a complete denture (Figure 8). Maintenance of good oral hygiene was emphasized. As part of the treatment process, genetic counselling was also provided to the patient and his parents. Additionally, a dermatologist was consulted for the management of the skin symptoms. Oral retinoids and skin emollients were prescribed for seven days. Regular monthly appointments were scheduled for proper follow-up.

## Discussion

PLS is an extremely rare genetic disorder that often goes undiagnosed until the patient develops a severely affected periodontium, thereby creating a significant social and psychological impact on the person diagnosed with the syndrome. The exact cause of PLS remains uncertain, but the development and progression of the syndrome are believed to be influenced by factors such as microbial, immunological, and mainly genetic factors. PLS has its primary gene locus on chromosome 11q14.1-q14.3. In homozygous PLS patients with cathepsin C gene mutations, loss of function has been identified [4]. This gene is abundantly expressed in immune defence cells and epithelial tissues, particularly in gingiva and the ventral skin surfaces of hands as well as feet [5]. Toomes et al. believed PLS had a strong genetic basis and their research has shown that patients with PLS exhibited amorphic mutations that affected the alleles of the cathepsin C gene, a lysosomal protease [6]. Cathelicidin antimicrobial peptide LL-37, which was found diminished in the gingival crevicular fluid and neutrophils of PLS patients, is produced by proteinase 3 and is essential for the production of cathepsin C [5].

Palmar-plantar keratosis, which frequently coexists with severe gingivitis of primary teeth, fast periodontal deterioration, and premature exfoliation of deciduous teeth at roughly four to five years of age, is what distinguishes PLS from other conditions. The same sequence of events is found to be recurring when permanent dentition erupts. By the age of 16, most permanent teeth may fall out similarly if they are not treated on time [2]. Patients with PLS may also have impaired neutrophil, lymphocyte, or monocyte function, increasing their vulnerability to bacterial infections and their risk of developing recurring pyogenic illnesses including pyoderma, furunculosis, pneumonia, and hepatic abscesses [4]. The development of periodontal pathogenic microorganisms may be explained by the reduced cytotoxic lymphocyte function seen in PLS patients [5]. Almuneef et al. conducted observations and concluded that pyogenic liver abscess can be recognised as a complication due to PLS as a result of compromised immune system function [7]. The presented case did not exhibit any abnormal liver function or ultrasonographic findings. However, ectopic calcifications were observed in the choroid plexus and falx cerebri, along with a delay in somatic development [4]. Gorlin et al., in their clinical and genetic analysis of 46 extensively documented PLS cases, proposed that the syndrome should be considered a triad of significant clinical features by adding calcification of the dura to palmar-plantar hyperkeratosis and premature periodontoclasia [8].

Early identification of PLS and a multidisciplinary approach can effectively improve patient prognosis. Systemic antibiotics are effective in reducing active periodontitis, while oral prophylactic measures can enhance the quality of life [5]. In a case report, Pacheco et al. concluded that scaling, root planing along with systemic drug therapy of amoxicillin-metronidazole effectively halts the progression of periodontal disease in individuals with PLS. This positive outcome could potentially be attributed to the alleviation or complete elimination of A. actinomycetemcomitans, as well as cytomegalovirus (CMV) and Epstein-Barr virus (EBV) [9]. Deciduous teeth with poor prognosis are removed, along with the eradication of periodontal infections, which can make it favourable for the erupting permanent teeth [10]. The choice of rehabilitation techniques for an edentulous PLS patient depends on various factors, such as the patient's specific requirements and preferences, support from surrounding bone structure, and the cost of treatment procedures. Treatment modalities including overdentures, conventional, modified and implant-supported complete dentures or a customised modification of these approaches may be advised for rehabilitation [10]. In a case report, Jain et al. highlighted that PLS has profound effects on individuals, causing social, psychological, and physical debilitation. They emphasized the importance of oral rehabilitation for such patients [11]. Prosthodontic rehabilitation plays a crucial role by providing a significant psychological boost to both the patient and their parents. It restores the aesthetic appearance and also enhances overall oral function [11]. Prosthetic rehabilitation in PLS may be tailored specifically to the patient's age and individual needs. It may involve initial rehabilitation using a complete or partial denture, with the option for considering implant-supported prostheses in the future [11].

To address skin lesions, treatment options such as emollients, salicylic acid, and urea can be utilized, while oral retinoids like acitretin and isotretinoin have demonstrated effectiveness in treating keratoderma [1]. Genetic counselling should be provided, and consanguinity should be discouraged to avoid inherited conditions reducing the quality of life of offspring. Parents of a child with PLS should be informed of the 25% chance of PLS in subsequent offspring [12].

## Conclusions

PLS is a highly uncommon inherited disorder that is autosomal and recessive in nature. The disorder has long-lasting, major effects on growing children's social, psychological, aesthetic, and functional characteristics. The etiopathogenesis of PLS, along with the dermatologic symptoms and periodontal implications that are unique to PLS, must be thoroughly understood by dental experts, to diagnose the condition in its earliest stages and to deliver appropriate and comprehensive dental care to the patient to restore their function and appearance and thus improve their quality of life. A multidisciplinary approach involving a periodontist, paediatrician, dermatologist, and prosthodontist is essential for the therapy of this disorder, with an insistence on genetic counselling for the affected person, dental and dermatological management, and psychological support.
